# Immune surveillance is thwarted by tumor-cell intrinsic beta-catenin signaling

**DOI:** 10.1186/2051-1426-3-S2-P418

**Published:** 2015-11-04

**Authors:** Stefani Spranger, Brendan Horton, Thomas F Gajewski

**Affiliations:** 1 University of Chicago, Chicago, IL, USA; 2 The University of Chicago School of Medicine, Chicago, IL, USA

## 

Growing evidence has emerged that subgroups of cancer patients have a spontaneous T cell-centered immune response reflected by infiltration of antigen-specific CD8^+^ T cells into the tumor microenvironment. However, another major subgroup of patients completely lacks T cell infiltration. Importantly, the presence of tumor-infiltrating CD8^+^ T cells has been correlated with clinical response to anti-PD-1 mAb and other immunotherapies. Recent work from our laboratory has revealed that activation of the Wnt/β-catenin pathway within tumor cells can mediate exclusion of T cells from the tumor microenvironment. Using an autochthonous inducible mouse model for melanoma (Braf^V600E^/PTEN^-/-^; BP) with or without stabilized β-catenin (Braf^V600E^/PTEN^-/-^/CAT-STA; BPC) we showed that T cell exclusion from the tumor microenvironment was due to failed recruitment of Batf3-lineage dendritic cells, resulting in a defective early T cell priming and absence of systemic immunity. However, whether tumor-intrinsic β-catenin signaling might mediate tumor resistance after an anti-tumor T cell response was established is not known. To test this notion, we used a spontaneously rejected cell line (MC57.SIY) to induce immunologic memory. These tumors were implanted into naïve BP and BPC mice that had been crossed to a Rosa26-LSL-SIY inducible antigen mouse and following complete regression of the primary (MC57.SIY) tumor, the genetically-induced melanomas were initiated. Although the primary SIY-specific CD8^+^ T cell response and the induced memory response were comparable between both tumor models, tumor protection was observed against BP-SIY tumors but not BPC-SIY tumors. Increased tumor control in BP-SIY mice was accompanied by strong T cell infiltration and a boosted memory response. These results indicate that tumor-intrinsic β-catenin signaling can mediate exclusion of effector T cell migration. To test this notion directly, we adoptively transferred in vitro-activated SIY-reactive T cell receptor transgenic T cells (2C T cells) into BP-SIY and BPC-SIY tumor-bearing hosts. In fact, primed 2C cells were only found in BP-SIY tumors but not BPC-SIY tumors. These results were confirmed using in vivo 2-photon microscopy of the tumor microenvironment. Taken together, these data provide strong evidence that up-regulation of tumor-intrinsic β-catenin is a strong mechanism of immune evasion even in the presence of immunologic memory. As such, mechanisms of immune surveillance and editing of tumors expand beyond antigen-loss but additionally via specific up-regulation of oncogene pathways. Moreover, tumor-intrinsic β-catenin activation likely can mediate resistance not only to checkpoint blockade but also T cell adoptive transfer.

